# Reshaping the Ability–Strategy Link in Emotion Regulation: The Role of a Structured Picture-Book Intervention for Preschoolers

**DOI:** 10.3390/bs15081137

**Published:** 2025-08-21

**Authors:** Lihong Wang, Ran Cui, Na Wan, Wei Hu

**Affiliations:** 1 Faculty of Education, Tianjin Normal University, Tianjin 300387, China; wanglihong@tjnu.edu.cn (L.W.); rc1526003127@163.com (R.C.); 2 School of Aerospace, Nanchang Institute of Technology, Nanchang 330044, China; 2013485@nut.edu.cn; 3 Department of Psychology, Fudan University, Shanghai 200433, China; 4 Key Research Base of Humanities and Social Sciences of the Ministry of Education, Research Institute of Psychology and Behavior, Faculty of Psychology, Tianjin Normal University, Tianjin 300387, China

**Keywords:** emotion-regulation ability, emotion-regulation strategy, ability–strategy relationship, structured picture-book intervention, preschoolers

## Abstract

Emotion-regulation ability and strategy (i.e., the specific behaviors used to manage feelings) are crucial for preschoolers’ socioemotional development. This study investigated whether a structured picture-book intervention could enhance these components and, critically, reshape the relationship between them. A quasi-experimental, pretest–posttest design was employed with 60 preschoolers (aged 4–5) assigned to an intervention or a passive-exposure control group. The intervention group engaged in bi-weekly, structured emotion-themed picture-book activities for eight weeks. Results from repeated-measures analyses indicated that the intervention group showed significantly greater gains in emotion-regulation abilities (i.e., recognition, expression, regulation) and more frequent use of positive strategies (e.g., cognitive reconstruction, seeking support) compared to the control group. Crucially, the intervention altered the relationship between ability and strategy. In the intervention group, the correlation between overall emotion-regulation ability and the use of negative strategies shifted from non-significant at pretest to significantly negative at posttest. Conversely, this relationship shifted to significantly positive in the control group. These findings suggest that structured interventions not only improve discrete emotion skills but also foster a more adaptive integration of ability and strategy use, preventing the maladaptive pattern where higher ability paradoxically links to greater reliance on negative strategies.

## 1. Introduction

Emotion regulation refers to a series of external and internal processes for monitoring, evaluating, and modifying emotional responses to achieve predetermined goals (Cole et al., 1994; Lazzarelli et al., 2024). Good emotion regulation is an important condition for ensuring young children’ mental health (Theodorou et al., 2024; Young et al., 2019; Quiñones-Camacho & Davis, 2018), facilitating normal interpersonal interactions (Zagaria et al., 2023), resolving peer conflicts (Ferro et al., 2023), and promoting better environmental adaptation (Hyson, 1990).

Ages 4–5 represent a critical period for children’s emotion ability development, when neural synapses in the brain continuously increase, and the amygdala and prefrontal cortex related to emotion regulation develop rapidly (e.g., Silvers et al., 2017; Goodpaster et al., 2025). Children at this stage have already acquired the ability to understand basic emotions. Simultaneously, the rapid development of language, theory of mind, and executive functions provides an important neurophysiological foundation for the enhancement of emotion regulation (Zheng & Ma, 2009; Jones & Bouffard, 2012).

Picture-book reading is an important vehicle for preschoolers’ cognitive development and a widely recognized and effective method for their emotion education (e.g., Chen & Huang, 2025; Wang & Shao, 2025). Picture books can display the complete process of emotion behavior through carefully designed environments, distinctive character images, and complete storylines, which greatly benefit preschoolers’ learning of emotion regulation (Yang & Lin, 2022). Picture-book thematic activities are one of the common teaching methods for emotion education, which include not only picture-book teaching and reading but also a series of enriching extension activities such as interactive discussions, experiential games, creative writing and drawing, and artistic performances (e.g., LaForge et al., 2018; Schoppmann et al., 2023; Britt et al., 2016; López & Friedman, 2019). Through these extension activities, children internalize the knowledge conveyed in picture books.

### 1.1. The Interplay of Emotion-Regulation Ability and Strategy: A Theoretical Framework

Emotion regulation is a multifaceted construct, encompassing not only the specific actions individuals take to manage their feelings, but also the underlying capacities that enable these actions. To understand its development, it is crucial to distinguish between two core components: emotion-regulation ability and emotion-regulation strategy. Ability refers to a higher-order, cognitive-emotional capacity, including the awareness and understanding of one’s own and others’ emotions (e.g., recognizing that a quivering lip may signify sadness), distress tolerance, and the clarity to identify emotional states (Gratz & Roemer, 2004; Hofmann & Kashdan, 2010). Strategy, in contrast, refers to the specific behavioral or cognitive tactics employed in a given situation to modulate an emotional response, such as taking deep breaths when angry (i.e., a behavioral strategy) or cognitive reappraisal, problem-solving, or distraction (Gross, 2015; Koole, 2009).

While it may seem intuitive that ability influences strategy choice, the relationship between these two components is not merely unidirectional. A more nuanced perspective is offered by Tull and Aldao’s (2015) bidirectional interactive model, which serves as the theoretical framework for the present study. This model posits that ability and strategy are mutually constitutive and engaged in a dynamic feedback loop. On one hand, an individual’s regulatory ability shapes which strategies they adopt and how effectively they are implemented. For instance, greater emotional clarity may facilitate the selection of more context-appropriate strategies. On the other hand, the repeated use of specific strategies can, in turn, strengthen or erode regulatory ability over time. The habitual use of adaptive strategies like cognitive reconstruction may enhance one’s overall sense of emotional efficacy, whereas chronic reliance—defined as the repeated, inflexible use of certain strategies even when they are ineffective (Coifman & Summers, 2019; Battaglini et al., 2022)—on maladaptive strategies like suppression can diminish emotional clarity. This dynamic interplay can create either adaptive or maladaptive cycles, fundamentally shaping an individual’s long-term emotional development.

This bidirectional model is particularly relevant for understanding the impact of interventions. It suggests that a successful intervention should not only enhance abilities and teach strategies in isolation but should aim to cultivate a more adaptive, synergistic relationship between them. The present study is grounded in this framework, seeking to investigate whether a structured intervention can actively reshape this ability–strategy link in preschoolers.

### 1.2. Effects of Picture-Book Interventions on Emotion-Regulation Ability

A growing body of research supports the view that structured picture-book reading interventions can effectively enhance preschoolers’ emotion-regulation abilities, especially when these interventions are grounded in developmental theory and implemented through intentional adult guidance and activity design. For instance, LaForge et al. (2018) developed six picture books based on Pons and Harris’s developmental model of emotion understanding, targeting early-stage competencies such as emotion recognition, external causes, and internal states like desires and beliefs. In a controlled experiment with preschoolers aged 34–54 months, children in the experimental group—who were repeatedly exposed to these theory-based books—showed significant gains in their Test of Emotion Comprehension (TEC) scores compared to the control group. While this study primarily focused on emotion understanding, the structured nature of the intervention offers a theoretical foundation for its potential to support later regulatory outcomes. Tsai (2008) further examined this issue using a quasi-experimental design involving 5- and 6-year-old Taiwanese kindergarteners. Two experimental groups were compared: one received emotion-related picture books with alphabet-based activities, and the other received the same books with targeted emotion-focused activities. Compared to the control group (which received animal-themed books and alphabet activities), both experimental groups demonstrated significantly greater improvements in emotion regulation, as measured by the Emotion Regulation Checklist (ERC), although only the group receiving emotion-focused activities showed superior performance in emotion understanding. Interestingly, no group differences were found on the parent-reported Head Start Competence Scale, suggesting that the effects of structured interventions may manifest more clearly in teacher-observed behaviors within educational settings.

Recent studies have reinforced and extended these findings. A randomized controlled study by Bergman Deitcher et al. (2020) showed that a small-group intervention using Ellis’s ABC model significantly improved preschoolers’ ability to express emotions and connect thoughts to feelings. The importance of specific interactive techniques has also been highlighted; Nurvinkania and Salim (2024) found that a dialogic reading intervention significantly improved children’s emotional understanding, with WH- and open-ended questions proving particularly effective.

Complementing these quantitative findings, observational and qualitative research provides deeper insights. An observational study by Ng and Sun (2022) in Singapore classrooms showed that shared book reading facilitates children’s ability to identify and label emotions in practical social contexts. From a micro-cognitive perspective, Dermata (2019) provided qualitative insights into the micro-cognitive processes involved. Her study showed that preschoolers rely heavily on non-verbal cues such as facial expressions and body posture when interpreting emotion content in picture books. However, their understanding of more abstract emotion constructs often remains concrete and self-centered. This indicates that structured guidance is essential not only for emotion comprehension but also for fostering the internalization of regulatory strategies, especially when engaging with multimodal emotion cues.

### 1.3. Effects of Picture-Book Interventions on Emotion-Regulation Strategy

While these studies collectively demonstrate that structured picture-book interventions can significantly enhance emotion-regulation abilities in early childhood, an important question concerns how such interventions may influence the use and refinement of specific emotion-regulation strategies—a topic that has received increasing empirical attention. For instance, one study found that even 22-month-old toddlers can effectively learn the emotion-regulation strategy of distraction by observing a model in a structured situation. This learning occurred regardless of whether the model was a familiar parent or an unfamiliar experimenter, highlighting the power of direct observation (Schoppmann et al., 2019). Further investigation with 24-month-olds confirmed that toddlers learn distraction through observation and that using this strategy helps reduce their negative affect. While toddlers’ individual temperaments (such as activity level) might influence their choice of a specific distraction activity (active vs. calm), this temperamental preference does not determine the overall effectiveness of learning the strategy from a model (Schoppmann et al., 2022). Building on this, other forms of learning, like picture-book thematic activities, also improve preschoolers’ emotion-related strategy use. Recent research found that picture-book reading, especially when delivered interactively, can help 3-year-old children adopt age-appropriate emotion-regulation strategies such as distraction. In waiting situations designed to elicit negative affect, children who read picture books featuring a protagonist using distraction showed increased use of this strategy compared to those in a control condition, particularly when the book was read interactively (Schoppmann et al., 2023).

While laboratory-based studies like Schoppmann’s demonstrate that specific strategies can be taught to toddlers and younger children, they establish a crucial foundation, suggesting that older preschoolers with more developed cognitive abilities are likely even more receptive to such learning. It is also crucial to understand the natural developmental trajectory of strategy use that interventions must align with. For instance, Sala et al. (2014) found that the variety of emotion-regulation strategies increases significantly with age between 3 and 6 years, with 5–6-year-olds showing a broader repertoire—including the emergence of cognitive reappraisal—compared to 3–4-year-olds. In contrast, Stansbury and Sigman (2000) reported that 3-year-olds, in certain frustration contexts, used more instrumental strategies than 4-year-olds, and that even at age 3, some children employed cognitive reappraisal. These differences highlight that while specific strategy distributions may vary across studies and contexts, both findings underscore that the preschool years are a dynamic period of change in strategy diversity and sophistication. Zhang et al. (2012) examined 3–5-year-old preschoolers’ understanding of fear emotions and their regulation strategies to analyze emotion responses and coping methods when facing real and imaginary fear sources at different ages. Researchers examined preschoolers’ fear experiences and regulation strategies under different fear sources (real fear objects (sharks, snakes, and tigers) or imaginary fear objects (ghosts, witches, or monsters)) and situations (4 real situations and 4 imaginary situations). The study found that 3-year-olds feared real situations more, 4-year-olds showed more significant fear of imaginary fear sources, and 5-year-olds used a greater variety of regulation strategies; preschoolers’ understanding of fear emotions varied significantly across different situations (alone, with parents, with peers). With increasing age, preschoolers’ understanding of fear emotions gradually strengthened, and regulation strategies shifted from reliance on external support to internal regulation.

### 1.4. Research Gaps

A growing body of research has begun to illuminate the crucial link between preschoolers’ emotion-regulation abilities and their use of specific strategies. These investigations have established that children’s understanding of different emotions influences their choice of coping tactics. For example, the study by Davis et al. (2010) found that children employ differentiated regulation strategies when facing different emotions: they tend to use goal reinstatement and agent-focused tactics rather than metacognitive strategies when angry; primarily modify their thoughts when frightened; and more frequently adjust their goals when sad. This suggests that children’s selection of emotion-regulation strategies is based on their recognition and understanding of emotion categories. Dennis and Kelemen (2009) explored how preschool children understand emotion-regulation strategies. The research included 62 children aged 3–4 years who watched puppet scenarios depicting characters experiencing negative emotions (anger, sadness, and fear), and evaluated the effectiveness of various emotion-regulation strategies. The findings revealed that the effectiveness of emotion-regulation strategies varied by emotion type. Repairing the situation was considered most effective for reducing anger, behavioral distraction and repairing the situation were considered more effective than cognitive distraction for reducing sadness, and behavioral distraction was considered more effective than cognitive distraction for reducing fear. Sala et al. (2014) also found a significant correlation between emotion understanding and emotion-regulation strategies. These investigations show that children start to distinguish between various approaches to managing emotions as a result of improvements in their understanding of how emotion events function. With this information, they can use tactics to alter situational antecedents and establish more favorable circumstances (Harris & Lipian, 1989).

However, while this work provides an important correlational foundation, its scope is limited in two key respects. First, being largely descriptive, these studies do not reveal whether the relationship between ability and strategy can be dynamically altered through a targeted intervention. Second, by often conceptualizing a unidirectional influence from ability to strategy, they do not fully account for the reciprocal dynamics proposed by contemporary theoretical models. The more complex, bidirectional relationship, as conceptualized by Tull and Aldao (2015), remains largely untested in an experimental context. This leaves a critical gap in the literature: Can a structured intervention transcend the mere enhancement of discrete skills to fundamentally reshape the functional interplay between regulatory ability and strategy use into a more adaptive system?

### 1.5. Present Study

The present study addresses this gap by employing a quasi-experimental, pretest-posttest control group design to examine how a structured, emotion-themed picture-book intervention impacts preschoolers’ emotion-regulation abilities, their use of strategies, and, most importantly, the relationship between the two.

The present study will primarily answer three scientific questions: (1) Whether picture-book thematic activities could significantly improve preschoolers’ emotion-regulation abilities? (2) Whether picture-book thematic activities could significantly improve preschoolers’ emotion-regulation strategies? (3) Whether picture-book thematic activities could significantly optimize the relationship pattern between preschoolers’ emotion-regulation abilities and strategies?

## 2. Materials and Methods

### 2.1. Participants

A public kindergarten in Tianjin was selected through convenience sampling for this study. This kindergarten was chosen due to its representative class structure and its willingness to cooperate with the research. The selection was contextualized within the local preschool education system. In Tianjin, preschools are guided by the Chinese Ministry of Education’s “Learning and Development Guide for Children Aged 3–6” (Ministry of Education of the People’s Republic of China, 2012) which emphasizes the importance of social-emotional development. However, systematic emotional curricula are not uniformly implemented in practice. While teachers receive general pedagogical training, specific training in children’s emotion-regulation strategies is limited, and daily classroom practices tend to focus more on cognitive and artistic domains. Within this context, the present study was designed to systematically evaluate an accessible and effective intervention strategy for preschool settings.

After obtaining approval from the kindergarten’s administration, informed consent forms were distributed to the parents of all age-eligible (4–5 years old) children. Parents of 60 preschoolers provided written informed consent. These children, from two existing classes, were assigned to either an intervention group (15 males, 15 females; average age 54 months) or a control group (17 males, 13 females; average age 53 months) via cluster randomization. An independent samples *t*-test confirmed no significant difference in age between the two groups (*p* > 0.05). Furthermore, according to the kindergarten’s records, the lead teachers of both classes did not significantly differ in terms of their teaching experience and professional levels, ensuring comparability between the groups. The intervention group participated in twice-weekly picture-book thematic activities, while all other activities remained the same as the control group.

The study was approved by the Institutional Review Board Research Institute of Psychology and Behavior, Tianjin Normal University (No. 2019050111).

### 2.2. Materials

Picture-book selection criteria were established based on literature (Tsai, 2008) and recommendations from preschool education experts: (1) Chinese picture books suitable for 4–5-year-old preschoolers’ psychological development; (2) picture books close to preschoolers’ lives and easily understood by them; (3) picture-book plots containing at least one common emotion experienced by preschoolers; (4) selected picture books with educational value for emotion regulation; (5) picture books with rich and interesting plots (see Table 1).

According to these criteria, 18 picture books were initially selected, then further screened through the “Chinese Local Emotion Picture Book Evaluation Questionnaire” by 20 preschool education professionals including researchers and kindergarten teachers. Finally, 8 Chinese picture books were selected as research materials, the consistency coefficient of the evaluators’ reliability is 0.87. The reading difficulty appropriateness of each selected picture book were also evaluated on a five-point scale (1 being very unsuitable, 5 being very suitable). Each picture book scored above 4.

### 2.3. Measures

The “Children’s Emotional Development Scale” compiled by Kuo and Teng (2021) and the “Preschool Children’s Emotional Regulation Strategies Inventory” compiled by Lu and Chen (2009) were used to assess preschoolers’ emotion-regulation abilities and use of emotion-regulation strategies, respectively, to explore the intervention effects of picture-book thematic activities using emotion-themed picture books as carriers.

The Children’s Emotional Development Scale was completed by the lead teacher of each child, based on their daily observations. It includes four dimensions: emotion recognition and understanding (the ability to perceive and identify one’s own and others’ emotional states and understand the causes of these states; e.g., “While listening to a story, can sense the characters’ joy, anger, sadness, or fear”), emotion expression (expressing emotions through spoken or written language, body movements, etc.; e.g., “When feeling angry, can accurately express it with appropriate words such as ‘angry,’ ‘mad,’ or ‘furious’”), emotion regulation (using certain strategies to restore emotional balance; e.g., “When feeling low, responds positively to an adult’s comfort”), and emotion application (applying appropriate emotion-regulation methods to others to help them cope with negative emotions; e.g., “When a peer is upset, shows understanding and sympathy, and engages in helping behaviors”). The scale contains a total of 21 items. It uses a five-point scoring method to examine the frequency of occurrence of various emotional regulation abilities, with “Always” scored as 5 points and “Never” scored as 1 point. The internal consistency reliability coefficient of this questionnaire is 0.94.

The Preschool Children’s Emotional Regulation Strategies Inventory was completed by the parent of each child, based on their knowledge of the child’s typical behaviors. The questionnaire presents multiple scenarios with several possible responses, and parents rate the frequency with which their child would use each response on a five-point scale (1 = Never, 5 = Always). For example, in the scenario “While playing with peers, another child accidentally bumps into your child and causes mild pain,” possible responses include: (1) thinking it is okay because it was not intentional, (2) pushing the peer back, (3) moving away to avoid the peer, (4) feeling slightly upset but quickly resuming play, (5) crying immediately, and (6) reporting to the teacher. The inventory includes eight dimensions: cognitive reappraisal (viewing the event from a positive perspective), problem-solving (using one’s own or others’ resources to resolve the situation), seeking support (asking others for help), alternative activities (shifting attention to other things), self-comfort (reassuring oneself through words or actions), passive coping (avoiding or doing nothing in response), emotional venting (shouting, crying, or similar actions), and aggressive behavior (verbal or physical aggression toward others). The internal consistency reliability coefficient of this questionnaire is 0.87.

### 2.4. Procedure

First, the two questionnaires were used to conduct a pretest on the emotion-regulation levels of preschoolers in both the intervention and control groups. Afterward, picture-book thematic activity intervention was implemented for the intervention group preschoolers. Specifically, while ensuring that the intervention group preschoolers’ daily life routines remained basically unchanged, picture-book thematic activities were added. The intervention period lasted eight weeks, with a picture-book activity and a thematic extension activity using the same picture book conducted each week. The activity process included: (1) Introducing the emotion theme of the picture book through the cover pattern. (2) Children independently reading the picture book accompanied by music, feeling the emotion changes within. (3) Teachers and children co-reading the picture book, sorting through the picture-book plot, with children identifying and understanding emotion changes (accompanied by music). (4) Teacher–child and child–child discussions about emotion-regulation strategies. (5) Asking children to share their own emotion stories and correct resolution strategies. (6) Teachers summarizing emotion-regulation strategies. Activity extensions included creating emotion-regulation theme walls and role-playing games. Control group preschoolers maintained their original daily life routines, with emotion picture books only placed in the book area for preschoolers to read on their own, also accompanied by the same short instrumental, lyric-free background music used in the intervention condition to ensure consistency. After the intervention, the two questionnaires were used to conduct a posttest on the emotion-regulation ability and strategy of preschoolers in both the intervention and control groups.

### 2.5. Researcher’s Role and Intervention Fidelity

Throughout the eight-week intervention, one of the researchers acted as a teaching assistant in the intervention classroom. The researcher’s primary responsibilities included preparing the picture books and materials for each session, assisting the lead teacher in managing group discussions, and observing and recording children’s reactions and verbal contributions. To minimize potential researcher bias, the core instructional tasks, such as storytelling and initiating key questions, were carried out by the class’s lead teacher. The researcher did not lead the activities. In the control group, the researcher’s presence was limited to the pretest and posttest data collection periods.

To ensure that the intervention was implemented as designed, several fidelity measures were employed: (1) Standardized Protocol: A detailed intervention manual was provided to the lead teacher of the intervention group. This manual specified the objectives, procedures (e.g., introducing the theme, reading, co-reading), key guiding questions, and timing for each of the sessions across the eight weeks, consistent with the description in Section 2.4. (2) Teacher Training: Prior to the intervention, the researcher conducted a two-hour training session with the lead teacher. The training covered the contents of the manual, the theoretical basis of emotion regulation, and techniques for facilitating child-led discussions and summarizing strategies. (3) Adherence Monitoring: The researcher’s presence as a teaching assistant allowed for direct monitoring of implementation. An implementation checklist, derived from the intervention manual, was used during each session to verify that all six core procedural steps were completed as planned. (4) Regular Debriefing: Weekly 15 min debriefing meetings were held between the researcher and the lead teacher to discuss session delivery, child engagement, and any challenges encountered. This ensured that any deviations were addressed promptly and that consistency was maintained across all sessions.

### 2.6. Data Analysis

Given that this study employed a quasi-experimental design with class-based grouping, potential pre-existing differences between the intervention and control groups were a key consideration. To directly quantify the magnitude of change for each participant, the gain score (posttest score minus pretest score) was utilized as the dependent variable. This approach allows for a straightforward assessment of pre-post change.

Mixed-design repeated-measures ANOVAs were conducted for both emotion-regulation ability and emotion-regulation strategies, each with one within-subjects factor (sub-dimensions) and one between-subjects factor (group), using gain scores as the dependent variable. When the assumption of sphericity was violated, the Greenhouse–Geisser correction was applied, and original degrees of freedom were reported. Bonferroni correction was used for multiple comparisons to control Type I error, and partial *η*^2^ was reported as the effect size estimate.

In addition, a series of correlation analyses were conducted to examine the relationships between emotion-regulation abilities and strategies before and after the intervention, separately for the intervention and control groups.

## 3. Results

### 3.1. Comparison of Intervention Effects on Emotion-Regulation Abilities Between Intervention and Control Groups

A mixed-design repeated-measures ANOVA with one within-subjects factor (sub-dimensions of emotion-regulation ability) and one between-subjects factor (group) was performed with emotion-regulation ability level as the dependent variable.

**Figure 1:**
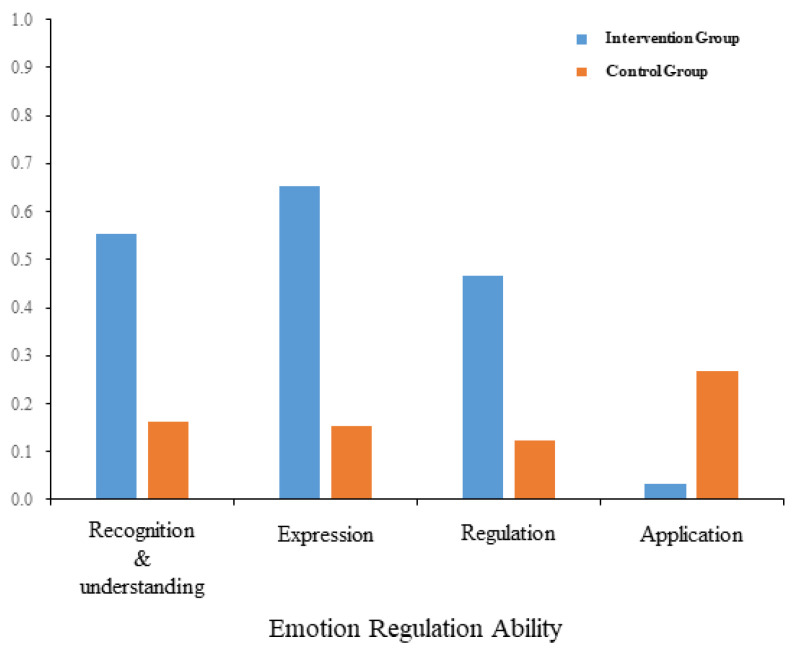
Intervention effects (posttest score minus pretest score) on emotion-regulation ability in the four sub-dimensions of recognition and understanding, expression, regulation, and application between the intervention and control groups.

Results showed a significant main effect among the gains in the four dimensions of emotion-regulation ability (emotion recognition and understanding, emotion expression, emotion regulation, and emotion application, see Figure 1) (*F*_3_,_174_ = 5.927, *p* = 0.001, *η*^2^_p_ = 0.093). Post hoc tests found that the gains in emotion recognition and understanding (0.3577 ± 0.4059) and emotion expression (0.4033 ± 0.5638) were significantly better than those in emotion application (0.1500 ± 0.4312, *p* = 0.001; *p* = 0.008), while emotion regulation (0.2945 ± 0.4382) showed no difference from the other three dimensions (*p*s > 0.05). The main effect of grouping was significant (*F*_1_,_58_ = 10.532, *p* = 0.002, *η*^2^_p_ = 0.154). Post hoc tests found that the intervention group’s gain (0.427 ± 0.055) was significantly higher than the control group’s (0.176 ± 0.055).

The interaction between emotion-regulation ability dimensions and grouping variables was significant (*F*_3_,_174_ = 13.214, *p* < 0.001, *η*^2^_p_ = 0.186). Simple effects showed that, on one hand, the intervention group performed significantly better than the control group in all four emotion-regulation ability dimensions (emotion recognition and understanding: *p* = 0.007; emotion expression: *p* < 0.001; emotion regulation: *p* = 0.027; emotion application: *p* < 0.001). On the other hand, in the intervention group, the scores for emotion recognition and understanding, emotion expression, and emotion regulation were all significantly better than those for emotion application (*p*s < 0.001), and emotion expression was significantly better than emotion regulation (*p* = 0.003), with no significant differences found in other pairwise comparisons (*p*s > 0.05). There were no statistical differences among the four sub-dimensions in the control group (*p*s > 0.05). Key statistical outcomes are summarized in Appendix A Table A1.

### 3.2. Comparison of Intervention Effects on Emotion-Regulation Strategies Between Intervention and Control Groups

The current analysis first combined the five emotion-regulation strategy sub-dimensions of cognitive reconstruction, problem-solving, seeking support, alternative activities, and self-comfort into positive emotion-regulation strategies, and the three emotion-regulation strategy dimensions of passive coping, emotional venting, and aggressive behavior into negative emotion-regulation strategies.

A mixed-design repeated-measures ANOVA with one within-subjects factor (sub-dimensions of emotion-regulation strategies) and one between-subjects factor (group) was performed with the use of emotion-regulation strategies as the dependent variable.

Results showed a significant main effect of emotion-regulation strategies (*F*_1_,_58_ = 6.560, *p* = 0.013, *η*^2^_p_ = 0.102). Post hoc tests found that the behavioral frequency gains of positive emotion-regulation strategies (0.077 ± 0.044) were significantly higher than those of negative emotions (−0.073 ± 0.045, *p* = 0.013). The main effect of grouping was not significant (*F*_1_,_58_ = 2.228, *p* = 0.141, *η*^2^_p_ = 0.037).

**Figure 2:**
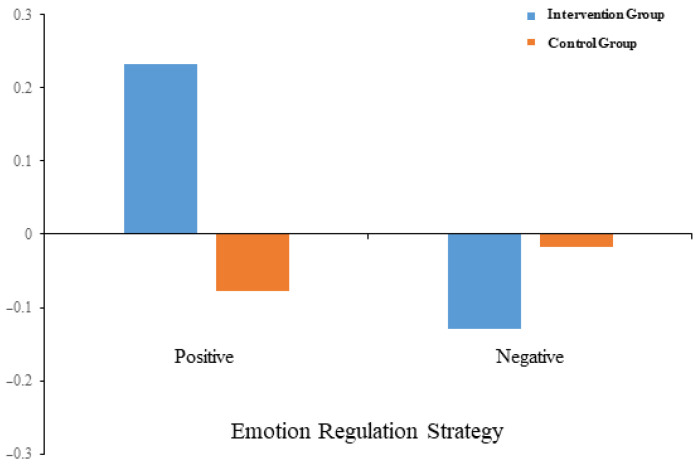
Intervention effects (posttest score minus pretest score) on emotion-regulation strategies in the positive strategy and the negative strategy use between the intervention and control groups.

The interaction between emotion-regulation strategy dimensions and grouping variables was significant (*F*_1_,_58_ = 13.024, *p* = 0.001, *η*^2^_p_ = 0.183). Simple effects showed that for positive emotion-regulation strategies, the intervention group performed significantly better than the control group (*p* = 0.001); for negative emotion-regulation strategies, the intervention group’s gain was lower than the control group’s, but the difference did not reach statistical significance (*p* = 0.214). See Figure 2.

**Figure 3:**
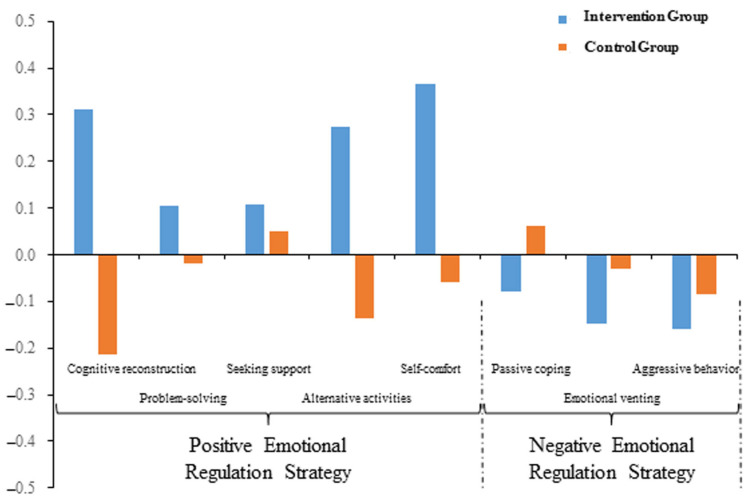
Intervention effects (posttest score minus pretest score) on emotion-regulation strategies in the five positive strategy dimensions of cognitive reconstruction, problem-solving, seeking support, alternative activities, and self-comfort, and the three negative strategy dimensions of passive coping, emotional venting, and aggressive behavior between the intervention and control groups.

Building on this, the study continued with a specific analysis of the sub-dimensions of emotion-regulation strategies. Results showed that all positive emotion-regulation strategies in the intervention group had positive values, indicating that posttest scores were better than pretest scores. However, the control group had both positive and negative values, indicating that subjects in the control group applied different positive emotion-regulation strategies relatively randomly. As for negative emotion-regulation strategies, the intervention group’s posttest scores were all better than (lower than) pretest scores. The control group’s application remained relatively random. Although the between-group differences were large, statistical tests showed that only the sub-dimensions of cognitive reconstruction (*p* = 0.001), alternative activities (*p* = 0.003), and self-comfort (*p* = 0.006) showed significant between-group differences. See Figure 3.

### 3.3. Comparison of Relationship Patterns Between Emotion-Regulation Abilities and Strategies Before and After Intervention in Intervention and Control Groups

**Figure 4:**
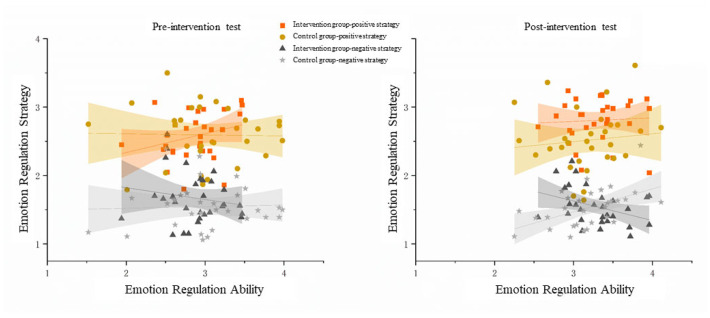
The correlation between the scores of emotion-regulation ability and positive emotion-regulation strategies or negative emotion-regulation strategies in the pre-intervention and post-intervention tests of both the intervention group and the control group.

A series of correlation analyses were conducted between emotion-regulation abilities and strategies before and after intervention in intervention and control groups (see Figure 4).

Pretest: In the intervention group, preschoolers’ emotion-regulation abilities were not significantly correlated with their positive emotion-regulation strategies (*r* = 0.277, *p* = 0.138) or with their negative emotion-regulation strategies (*r* = −0.168, *p* = 0.374); in the control group, preschoolers’ emotion-regulation abilities were not significantly correlated with their positive emotion-regulation strategies (*r* = −0.022, *p* = 0.906) or with their negative emotion-regulation strategies (*r* = 0.046, *p* = 0.811).

Posttest: In the intervention group, preschoolers’ emotion-regulation abilities were not significantly correlated with their positive emotion-regulation strategies (*r* = 0.059, *p* = 0.758) but were significantly negatively correlated with their negative emotion-regulation strategies (*r* = −0.376, *p* = 0.040). This indicates that under the influence of thematic picture-book activities, the more preschoolers’ emotion-regulation abilities improved, the less frequently they applied negative emotion-regulation strategies; in the control group, preschoolers’ emotion-regulation abilities were not significantly correlated with their positive emotion-regulation strategies (*r* = 0.111, *p* = 0.559) but were significantly positively correlated with their negative emotion-regulation strategies (*r* = 0.506, *p* = 0.004).

## 4. Discussion

This study aimed to examine the impact of thematic picture-book activities on the emotion-regulation ability and strategies of 4- to 5-year-old preschoolers, and to explore the dynamic changes in the relationship between them. Adopting a quasi-experimental design, the results showed that, compared to a control group serving as a passive exposure control, preschoolers in the intervention group demonstrated significant improvements in both their emotion-regulation ability and strategies following the picture-book thematic intervention. Most notably, the intervention altered the relational pattern between overall emotion-regulation ability and the use of emotion-regulation strategies. Specifically, in the intervention group, the relationship between overall emotion-regulation ability and the use of negative strategies shifted from being uncorrelated pre-intervention to significantly negatively correlated post-intervention. In the control group, however, this relationship also changed from uncorrelated to significantly positively correlated.

### 4.1. Intervention Effects on Emotion-Regulation Ability

In all four emotion-regulation ability dimensions (emotion recognition and understanding, emotion expression, emotion regulation, and emotion application), the intervention group’s intervention gains were significantly higher than those of the control group. In the intervention group, the scores for emotion recognition and understanding, emotion expression, and emotion regulation were all significantly better than those for emotion application; meanwhile, emotion expression was significantly better than emotion regulation. There were no differences among the four sub-dimensions in the control group.

In the dimensions of emotion recognition and understanding, emotion expression, and emotion regulation, intervention group preschoolers showed significant improvements, which is consistent with previous research findings (e.g., LaForge et al., 2018; Tsai, 2008). This indicates that after the intervention of emotion-themed picture-book thematic activities, preschoolers not only significantly improved in identifying and understanding others’ emotion states but also significantly enhanced their ability to communicate using emotion language, including verbal, facial expressions, and body movements. Additionally, their ability to control emotions was significantly strengthened. This may result from the design of Chinese picture-book thematic activity implementation, where activity plans particularly emphasized cultivating preschoolers’ emotion understanding and expression, encouraging them to share emotions they felt from stories and express their inner emotions and thoughts. Furthermore, role-playing activities also emphasized expressing emotions authentically through language and actions.

However, in the dimension of emotion application, the difference between pre- and post-tests for preschoolers in both the intervention and control groups did not reach significance. This may be because emotion application is considered an advanced skill in emotion regulation, involving helping others regulate negative emotions. For 4–5-year-old preschoolers, improvement in this dimension is relatively difficult (Vinden, 1999). This finding is consistent with recent research demonstrating that young children find it more challenging to respond to emotional needs than to instrumental ones. For example, a study by Karasewich et al. (2023) with 3.5- to 4.5-year-olds found significantly higher intervention rates in helping tasks (95.2% and 100%) than in comforting tasks (ranging from 88.1% for a broken toy down to just 40.5% for an injured person). This directly indicates that comforting behaviors, which are a core component of emotion application, are less prevalent in this age group than instrumental helping. This suggests that while our thematic activities were effective for foundational emotion skills, fostering advanced, pro-social regulatory abilities like helping others may require more targeted, long-term interventions, or may be more amenable to change in slightly older children with more developed theory of mind.

### 4.2. Intervention Effects on Emotion-Regulation Strategy

The research found that regarding intervention effects (posttest minus pretest), for positive emotion-regulation strategies, the intervention group performed significantly better than the control group; for negative emotion-regulation strategies, the intervention group’s gain was lower than the control group’s, but the difference did not reach statistical significance. This result stems from the incorporation of demonstrations of using positive strategies and analyses of the disadvantages of negative strategies in the picture-book thematic plan design, as well as guiding preschoolers to use positive strategies while avoiding negative strategies in extension activities. This is consistent with Schoppmann et al. (2023) study which demonstrates that preschoolers can learn specific emotion-regulation strategies through emotion-themed picture-book reading intervention.

Further analysis revealed that for positive emotion-regulation strategies, only in the sub-dimensions of cognitive reconstruction, alternative activities, and self-comfort did the intervention group perform significantly better than the control group. However, as shown in Figure 3, the intervention effects of the intervention group in all five positive strategy sub-dimensions (cognitive reconstruction, problem-solving, seeking support, alternative activities, and self-comfort) were positive values, meaning that posttest scores were better than pretest scores. It is noteworthy that the most significant gains were observed in strategies reflecting internal self-regulation (e.g., cognitive reconstruction, alternative activities, self-comfort), which align with reappraisal-type strategies described by Parkinson and Totterdell (1999). In contrast, strategies requiring more complex, cooperative problem-solving showed less improvement, which is developmentally appropriate for this age group and consistent with findings by Zhang et al. (2012). Specifically, the limited improvement in the ‘seeking support’ strategy can be understood through the lens of cooperative emotion regulation. A lack of seeking support is a primary behavioral sign of difficulty in this area, representing a failure to initiate co-regulation when needed. This can stem from a child’s lack of emotional awareness, an inability to articulate their needs, or a history of unresponsive caregiving (Zhao et al., 2025; also see Spilt et al., 2021). This underscores that seeking support is a complex, advanced competency, which may explain its resistance to change within the timeframe of our intervention.

On the other hand, for negative emotion-regulation strategies, the intervention group showed negative intervention effects in all three negative strategy sub-dimensions (passive coping, emotional venting, and aggressive behavior), showing that posttest scores were lower than pretest scores, which indicated the robustness of intervention activities. Control group preschoolers showed positive values for passive coping and negative values for emotional venting and aggressive behavior. This indicates that although control group preschoolers also gradually reduced their application of certain negative strategies, they still relied on specific negative strategies (e.g., passive coping).

### 4.3. Dynamic Link Between Emotion-Regulation Ability and Strategy

In the pretest, intervention group preschoolers’ emotion-regulation abilities were not correlated with either their positive or negative emotion-regulation strategies; similarly, control group preschoolers’ emotion-regulation abilities were not correlated with either their positive or negative emotion-regulation strategies. This result may reflect that before intervention, preschoolers’ overall emotion-regulation abilities had not yet developed to a stage where they could stably influence their specific strategy use. Previous research has shown that 4–5-year-old preschoolers’ emotion-regulation strategies are still in the early stages of development, and their strategy selection may be largely influenced by situation and external environment rather than stable individual abilities (Sabatier et al., 2017). Without systematic emotion-regulation training, preschoolers may still rely on intuitive or habitual ways to respond to emotion experiences, lacking clear strategic regulation.

In the posttest, both intervention and control group preschoolers’ emotion-regulation abilities remained uncorrelated with their positive emotion-regulation strategies. However, the intervention effect showed that intervention group preschoolers’ emotion-regulation abilities were significantly negatively correlated with their negative emotion-regulation strategies. That is, under the influence of thematic picture-book activities, stronger emotion-regulation abilities were associated with less use of negative emotion-regulation strategies. This pattern is consistent with evidence that insufficient emotion regulatory skills can directly precede negative social outcomes, whereas stronger emotional competence and regulation are linked to superior social skills, empathy, and problem-solving abilities (Cohen & Mendez, 2009; Luo et al., 2025). Furthermore, Korucu et al. (2022) found that in preschool children, the emotion-regulation factor was the primary predictor of social–emotional skills, underscoring the pivotal role of regulation in broader developmental outcomes. These findings can be interpreted through the theoretical framework of Tull and Aldao (2015), who proposed a bidirectional relationship model between emotion-regulation abilities and strategies. According to their model, emotion-regulation abilities are higher-order processes that influence the type of strategies an individual employs, while the repeated use of certain strategies may, in turn, enhance or diminish specific emotion-regulation abilities—creating either adaptive or maladaptive feedback loops. The significant negative correlation between emotion-regulation abilities and negative strategies observed in the intervention group exemplifies what Tull and Aldao would characterize as an adaptive feedback loop. As the intervention enhanced preschoolers’ emotion recognition, understanding, expression, and regulation abilities, these improved abilities likely enabled children to recognize the limited utility of negative strategies (passive coping, emotional venting, and aggressive behavior) and consequently reduced their reliance on such strategies. Simultaneously, the reduced application of negative strategies may have further reinforced their emotion-regulation abilities by creating more opportunities for successful emotion processing and resolution, thus establishing a positive, mutually reinforcing cycle.

Meanwhile, control group preschoolers’ emotion-regulation abilities were significantly positively correlated with their negative emotion-regulation strategies. That is, simply providing picture books for preschoolers to read independently not only failed to link improved emotion abilities with reduced negative strategies but actually resulted in stronger emotion-regulation abilities being associated with more use of negative strategies. This unexpected positive correlation in the control group also can be understood through what Tull and Aldao (2015) would describe as a maladaptive feedback loop. Without proper guidance in the implementation of emotion knowledge, preschoolers with stronger emotion awareness might actually become more sensitive to emotion challenges but lack the appropriate scaffolding to develop constructive responses. As a result, their heightened emotion recognition and understanding might lead to increased emotion reactivity without corresponding improvements in regulation strategies, pushing them toward negative coping mechanisms. This aligns with Tull and Aldao’s proposition that the relationship between abilities and strategies depends critically on environmental factors—in our case, structured picture-book intervention—that can either facilitate or hinder the development of adaptive emotion-regulation patterns. This finding is also similar to previous research: Vinden (1999) found that without structured emotion education, young children find it difficult to respond appropriately to others’ emotions. For example, when someone feels sad or distressed, they might respond with positive emotions to the person’s face. Evidently, relying solely on preschoolers’ independent exploration cannot guarantee effective learning of positive emotion-regulation strategies. During independent reading, preschoolers lack adult guidance to understand emotion-regulation situations in picture books and may even incorrectly reinforce certain negative coping methods (for example, if characters in stories exhibit anxiety or avoidance behaviors when facing challenges, preschoolers might imitate these negative strategies).

### 4.4. Limitations and Future Directions

This study adopted a quasi-experimental design, which, while increasing ecological validity, lacks the random assignment of a true experiment. Therefore, we cannot entirely rule out pre-existing group differences as a potential confounding variable. Future research should employ a randomized controlled trial (RCT) to establish causality more firmly.

A longitudinal study would be beneficial to track the long-term effects of the intervention and observe the stability of the changed ability–strategy relationship. Future studies should also investigate more effective intervention strategies for the ‘emotion application’ dimension. For instance, future interventions could incorporate specific modules on prosocial behavior, such as teaching children explicit strategies for comforting a peer. To this end, future research should systematically investigate factors such as age-related readiness for these advanced prosocial skills, the optimal duration and intensity of interventions, and the specific types of adult support (e.g., explicit instruction vs. guided co-construction of meaning) that most effectively nurture these more complex emotional competencies.

Finally, future research could benefit from multi-method assessments, combining teacher/parent reports with direct behavioral observations during emotion-eliciting tasks.

## 5. Theoretical and Practical Implications

This study demonstrates that a structured picture-book intervention does more than enhance discrete emotion-regulation skills in preschoolers; it actively reshapes the fundamental relationship between their regulatory abilities and strategy use. Our findings offer several key implications for developmental psychology and intervention science.

First, our results provide critical empirical support for a dynamic, bidirectional model of emotion-regulation development. The divergent trajectories of the ability–strategy relationship in the two groups illustrate that this link is not static but malleable. The intervention fostered an adaptive feedback loop, where enhanced ability was associated with reduced use of negative strategies. In contrast, passive exposure led to a maladaptive pattern, where gains in emotional awareness, without guided strategy development, paradoxically correlated with more negative strategy use. This highlights that the development of emotion regulation is an integrative process, and interventions must target not just the components but their functional integration.

Second, the findings underscore that the efficacy of such interventions hinges on the presence of an adult acting as an “emotion coach”. The stark contrast between the groups suggests that passive exposure to materials is insufficient. The structured activities in the intervention group created a context for the adult to validate feelings, help label emotions, and guide children in problem-solving and selecting appropriate strategies—core components of emotion coaching. This active, guided process is what appears to prevent the formation of maladaptive loops. For practitioners, this implies that the true agent of change is not the material itself, but the facilitated interaction around it.

Third, for this model to have a broader impact, its scalability and adaptability must be considered. In under-resourced settings, the principles of this intervention can be applied using low-cost, culturally relevant materials, such as local folktales or teacher-made storybooks. The “emotion coach” role can be integrated into daily routines rather than standalone lessons, maximizing existing teacher-child interaction time. A “train-the-trainer” approach could also be employed to disseminate the core skills efficiently among staff. For diverse cultural contexts, the intervention framework must be flexible, allowing for adaptation of the story content and emotional language to align with local values and norms, ensuring the concepts are both accessible and meaningful to children and educators.

Fourth, this research offers a new perspective for evaluating intervention efficacy. Traditionally, intervention success is measured by mean-level changes in target skills. Our findings suggest that a crucial, yet often overlooked, outcome is the change in the correlational structure among psychological constructs. Future intervention research in behavioral science could benefit from assessing not only whether skills improve, but whether they become more adaptively organized. This provides a more nuanced and psychologically meaningful index of developmental change. To advance this agenda, it is essential to consider the conditions under which such functional integration emerges. Future research should therefore systematically examine how the quality and depth of teacher training, as well as the moderating role of cultural context, shape not only skill acquisition but also the organization of competencies over time.

## 6. Conclusions

This study shows that structured thematic picture-book activities can effectively improve preschoolers’ emotion-regulation abilities and reduce their use of negative strategies. The intervention reshaped the link between ability and strategy, highlighting that guided support is essential for fostering adaptive emotion development. In contrast, passive exposure may be insufficient or even counterproductive, underscoring the importance of intentional, teacher-led implementation in early emotion education.

## Figures and Tables

**Table 1:** Details of emotional content and prompts in selected picture books.

No.	Book Title	Primary Ability Dimensions	Key Challenges & Situations	Core Emotions	Character’s Coping Strategies	Sample Teacher Prompts
1	*I Get Scared*	Emotion Recognition & Understanding	Experiencing fear from various stimuli; Feeling tension associated with fear	Fear	**Alternative activities**: Watching cheerful shows; **Self-comfort**: Hugging oneself	What makes Little Bear scared? What does being scared feel like? How does Little Bear make himself feel better?
2	*I Feel Sad*	Emotion Recognition & Understanding	Losing a favorite toy; General feelings of sadness and discomfort	Sadness	**Alternative activities**: Reading, playing with friends; **Emotional venting**: Crying	Why does Little Bear feel sad? What do you do when you feel sad? What does Little Bear do to feel better?
3	*I Get Mad*	Emotion Expression	Frustration from not getting what one wants; Realizing anger can negatively affect others	Anger	**Alternative activities**: Playing with other toys	What makes Little Bear angry? What methods did he use to stop being angry? What do you do when you are very angry?
4	*My Heart Goes Thump-Thump*	Emotion Expression	Facing performance anxiety; Worrying about failure and disappointing others	Nervousness, Fear	**Seeking support**: Telling a parent about feelings; **Self-comfort**: Taking slow, deep breaths; closing eyes to calm down	What is the child worried about? How does his mom comfort him? What does he do to calm his feelings?
5	*Throwing Away Bad Feelings*	Emotion Regulation	Encountering multiple frustrating events	Anger, Frustration	**Problem-solving**: Actively fixing issues; **Cognitive reconstruction**: Choosing to forgive and maintain a happy mood	What problem did Little Fox encounter? Did he get angry? How did he solve it? What do you do about things that make you unhappy?
6	*I Can Fix My Bad Feelings*	Emotion Regulation	Feeling disappointed by a broken promise; Not knowing how to manage strong anger	Anger	**Seeking support**: Asking others for their strategies for managing anger	What made Little Bull Lele very angry? What did he do to learn how to stop being angry? What do you do about things that make you unhappy?
7	*The Elephant Who Eats the Dark*	Emotion Application	Fear of the dark; Understanding unintended negative consequences of eliminating a negative emotion	Fear	**Cognitive reconstruction**: Realizing the necessity of night and that fear is a normal emotion; **Problem-solving**: Reversing a “solution” that caused new problems	How did the elephant help the animals? Why did his solution cause new problems? What did the animals learn about fear?
8	*Doudou Gets Angry*	Emotion Application	Anger from possessions being shared; Acting out aggressively; Causing fear and sadness in others	Anger, Fear, Sadness	**Self-comfort**: Taking deep breaths to calm down; **Seeking support**: Comforting the “hurt” toys to alleviate their sadness	Why is Doudou angry? How does he act? What methods does Doudou use to stop being angry? How can you help others understand your feelings?

Note. The dimensions presented are the primary focus, the source materials feature a broader range of themes.

## Data Availability

The data presented in this study are available on request from the corresponding author due to privacy and ethical reasons.

## Appendix A

**Table A1:** Summary of key statistical results.

Outcome Measure	Statistical Test	*F*-Value (df)	*p*-Value	*η* ^2^_p_	Key Results (M ± SD or *r*)
*EMOTION-REGULATION ABILITIES*					
*Main Effects & Interactions*					
*ER Dimensions (Within-subjects)*	Mixed ANOVA	*F*_3,174_ = 5.927	0.001 **	0.093	Recognition: 0.358 ± 0.406; Expression: 0.403 ± 0.564; Regulation: 0.295 ± 0.438; Application: 0.150 ± 0.431
*Group Effect (Between-subjects)*	Mixed ANOVA	*F*_1,58_ = 10.532	0.002 **	0.154	Intervention: 0.427 ± 0.055; Control: 0.176 ± 0.055
*Dimensions × Group Interaction*	Mixed ANOVA	*F*_3,174_ = 13.214	<0.001 ***	0.186	All dimensions: Intervention > Control
*Between-Group Comparisons*					
*Emotion Recognition & Understanding*	Simple Effects	-	0.007 **	-	Intervention > Control
*Emotion Expression*	Simple Effects	-	<0.001 ***	-	Intervention > Control
*Emotion Regulation*	Simple Effects	-	0.027 *	-	Intervention > Control
*Emotion Application*	Simple Effects	-	<0.001 ***	-	Intervention > Control
*EMOTION-REGULATION STRATEGIES*					
*Main Effects & Interactions*					
*Strategy Type (Positive vs. Negative)*	Mixed ANOVA	*F*_1,58_ = 6.560	0.013 *	0.102	Positive: 0.077 ± 0.044; Negative: −0.073 ± 0.045
*Group Effect*	Mixed ANOVA	*F*_1,58_ = 2.228	0.141	0.037	n.s.
*Strategy Type × Group Interaction*	Mixed ANOVA	*F*_1,58_ = 13.024	0.001 **	0.183	Positive strategies: Intervention > Control
*Strategy Sub-dimensions*					
*Cognitive Reconstruction*	Between-group	-	0.001 **	-	Intervention > Control
*Alternative Activities*	Between-group	-	0.003 **	-	Intervention > Control
*Self-comfort*	Between-group	-	0.006 **	-	Intervention > Control
*CORRELATIONAL RELATIONSHIPS*					
*Pretest Correlations*					
*ER Abilities × Positive Strategies (Intervention)*	Pearson’s r	-	0.138	-	*r* = 0.277 (n.s.)
*ER Abilities × Negative Strategies (Intervention)*	Pearson’s r	-	0.374	-	*r* = −0.168 (n.s.)
*ER Abilities × Positive Strategies (Control)*	Pearson’s r	-	0.906	-	*r* = −0.022 (n.s.)
*ER Abilities × Negative Strategies (Control)*	Pearson’s r	-	0.811	-	*r* = 0.046 (n.s.)
*Posttest Correlations*					
*ER Abilities × Positive Strategies (Intervention)*	Pearson’s r	-	0.758	-	*r* = 0.059 (n.s.)
*ER Abilities × Negative Strategies (Intervention)*	Pearson’s r	-	0.040 *	-	*r* = −0.376
*ER Abilities × Positive Strategies (Control)*	Pearson’s r	-	0.559	-	*r* = 0.111 (n.s.)
*ER Abilities × Negative Strategies (Control)*	Pearson’s r	-	0.004 **	-	*r* = 0.506

Note. * *p* < 0.05, ** *p* < 0.01, *** *p* < 0.001; n.s. = not significant; ER = emotion regulation.
